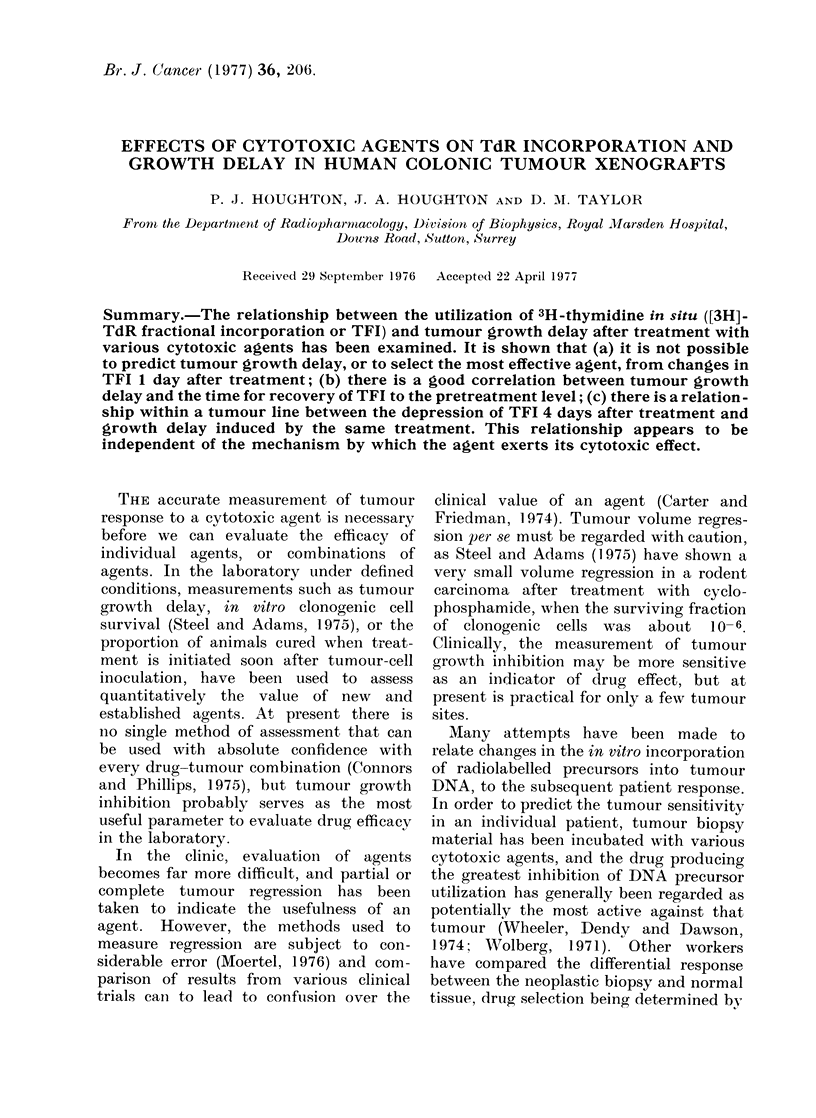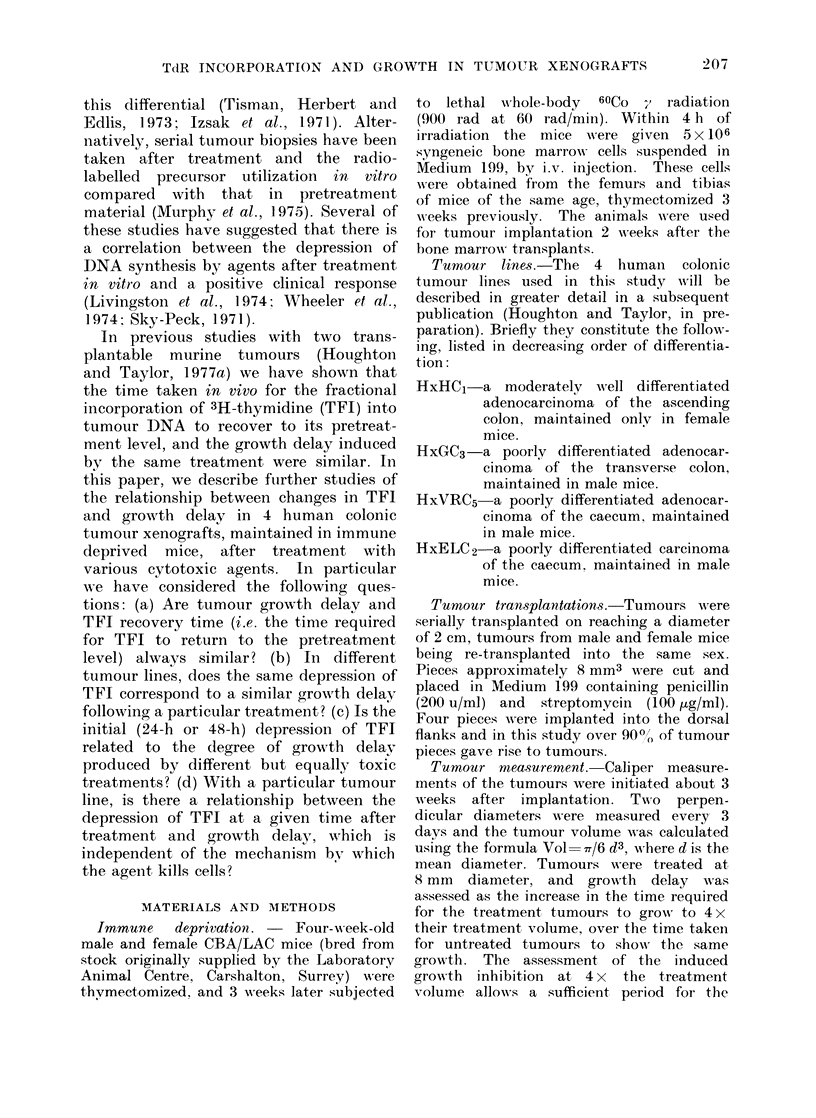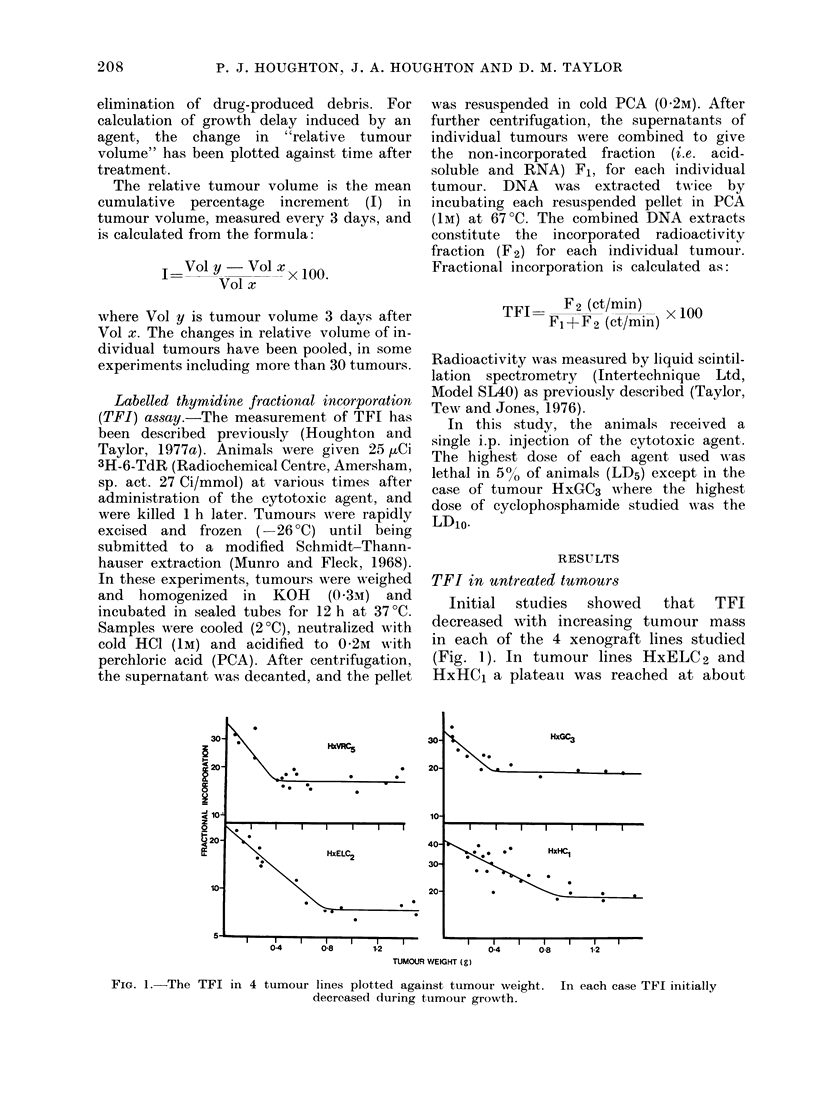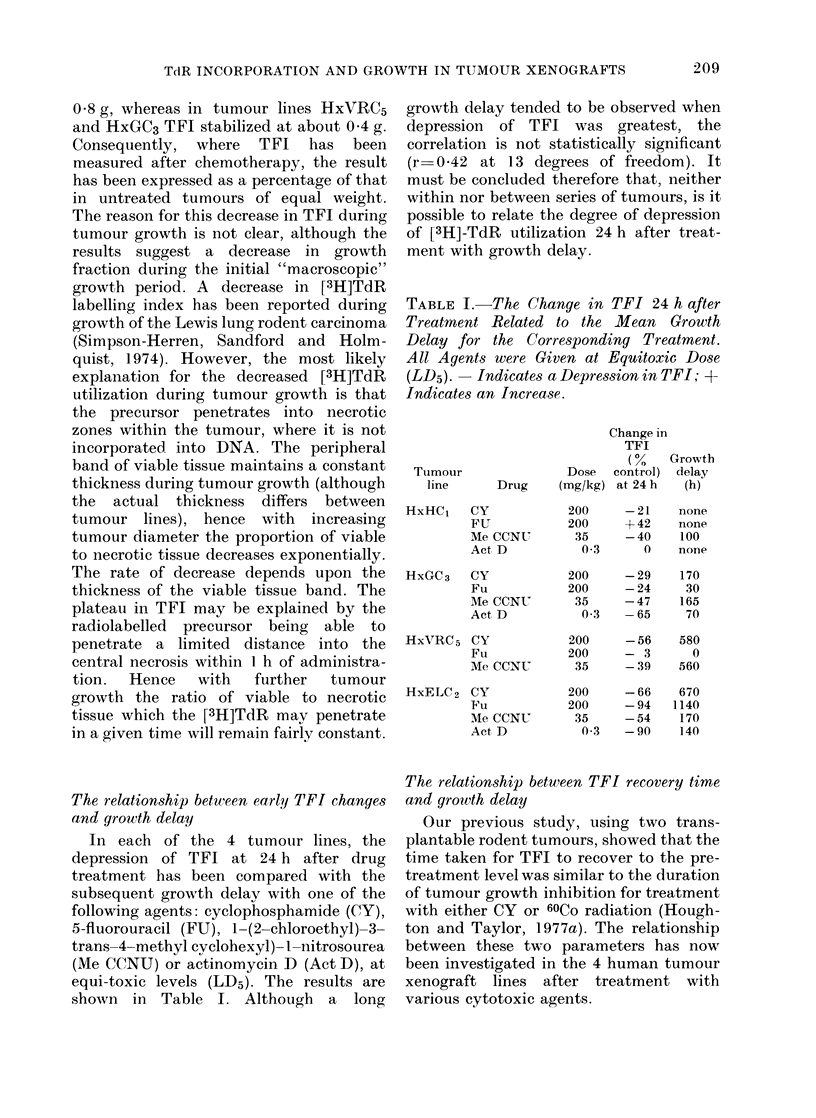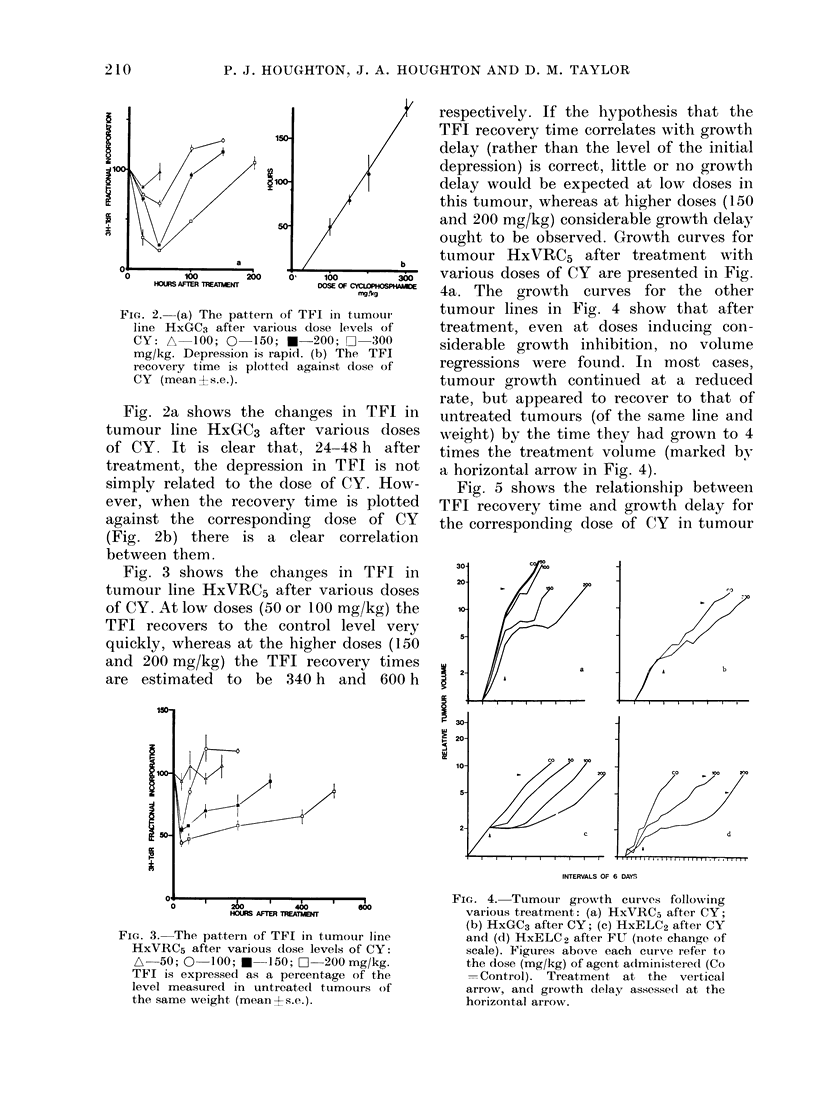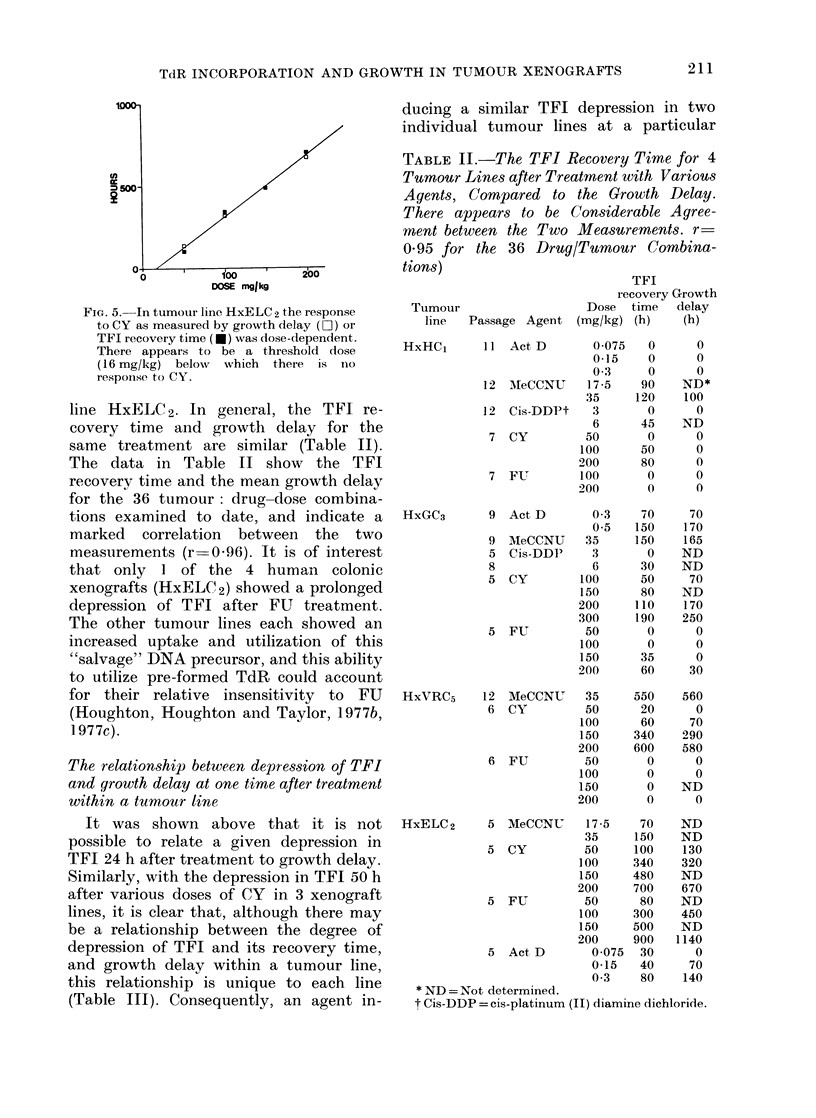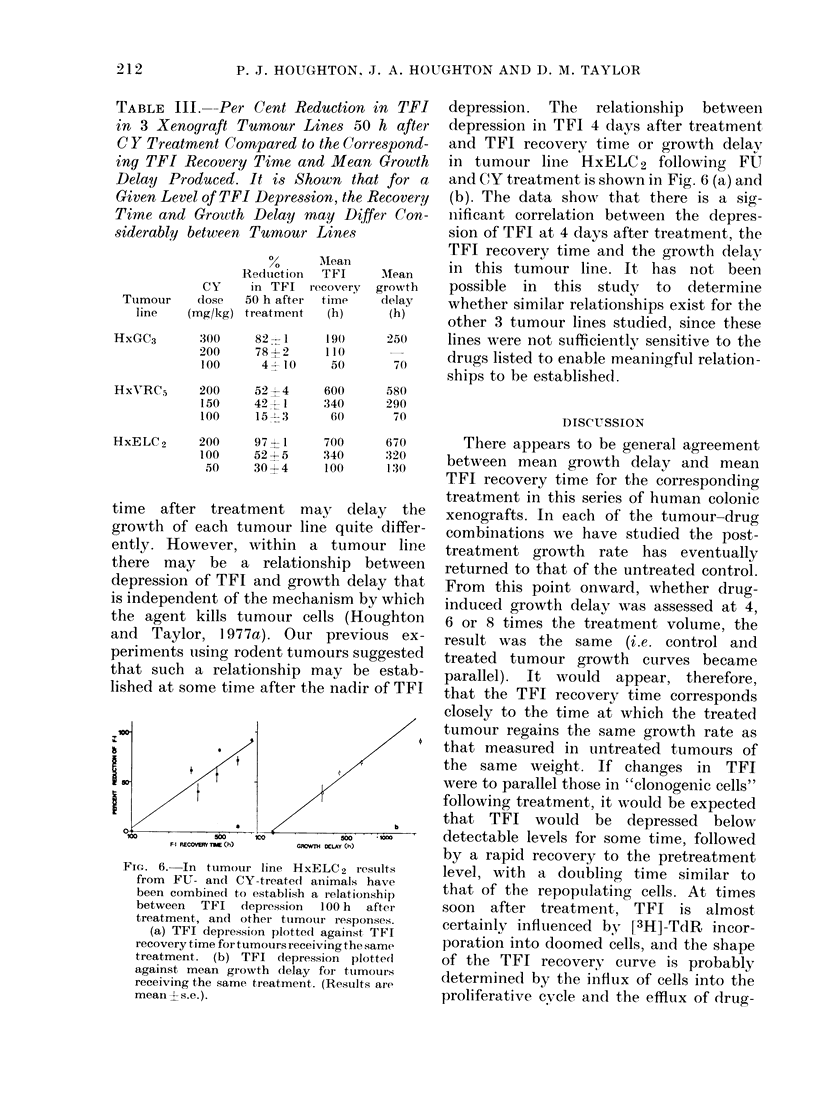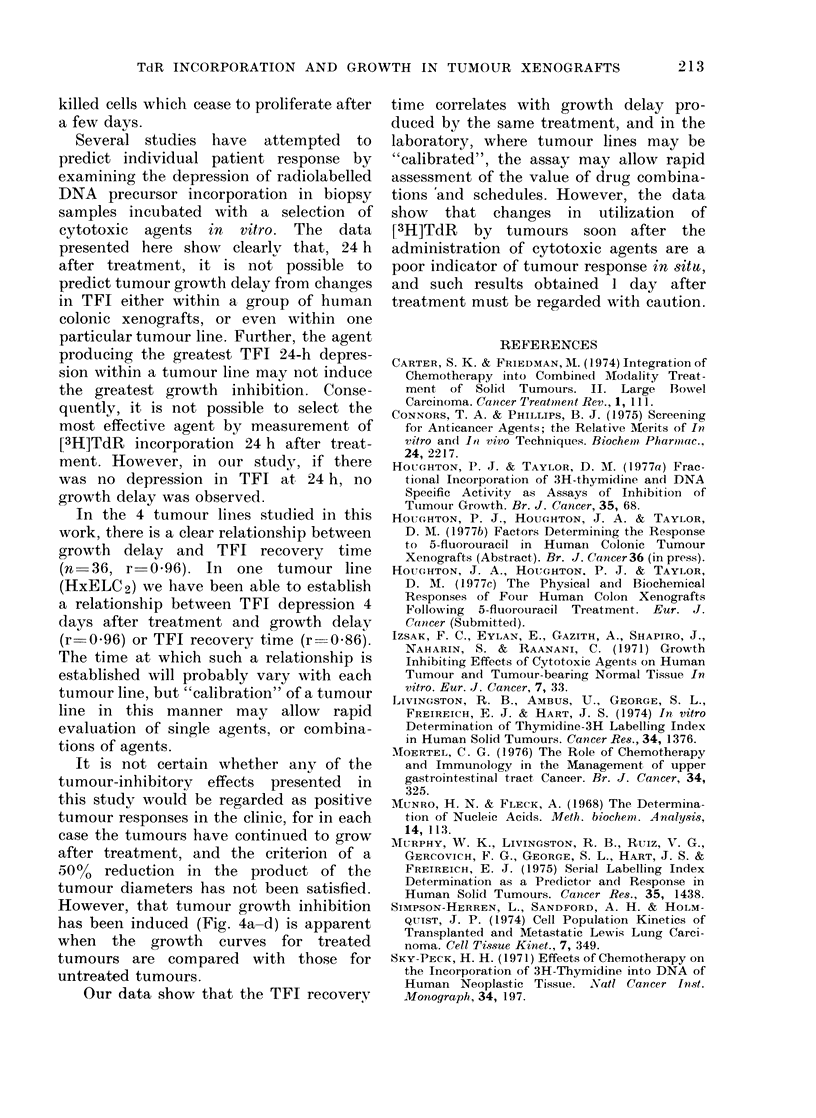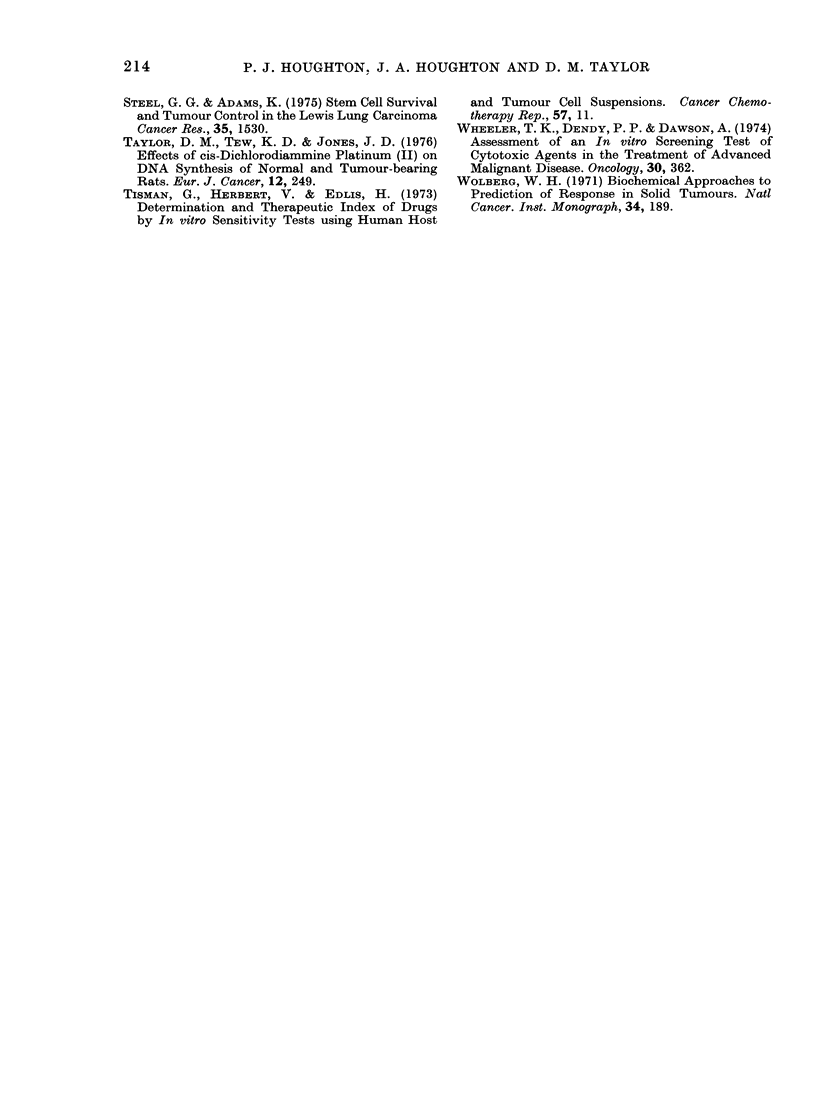# Effects of cytotoxic agents on TdR incorporation and growth delay in human colonic tumour xenografts.

**DOI:** 10.1038/bjc.1977.179

**Published:** 1977-08

**Authors:** P. J. Houghton, J. A. Houghton, D. M. Taylor

## Abstract

The relationship between the utilization of 3H-thymidine in situ ([3H]-TdR fractional incorporation or TFI) and tumour growth delay after treatment with various cytotoxic agents has been examined. It is shown that (a) it is not possible to predict tumour growth delay, or to select the most effective agent, from changes in TFI 1 day after treatment; (b) there is a good correlation between tumour growth delay and the time for recovery of TFI to the pretreatment level; (c) there is a relationship within a tumour line between the depression of TFI 4 days after treatment and growth dealy induced by the same treatment. This relationship appears to be independent of the mechanism by which the agent exerts its cytotoxic effect.


Br. J. Cancer (1977) 36, 206.

### EFFECTS OF CYTOTOXIC AGENTS ON TdR INCORPORATION AND GROWTH DELAY IN HUMAN COLONIC TUMOUR XENOGRAFTS

P. J. HOUGHTON, J. A. HOUGHTON AND D. M. TAYLOR

*From the Department of Radiopharmacology, Division of Biophysics, Royal Marsden Hospital, Downs Road, Sutton, Surrey*

Received 29 September 1976 Accepted 22 April 1977

**Summary.—The relationship between the utilization of $^{3}H$-thymidine *in situ* ([3H]-TdR fractional incorporation or TFI) and tumour growth delay after treatment with various cytotoxic agents has been examined. It is shown that (a) it is not possible to predict tumour growth delay, or to select the most effective agent, from changes in TFI 1 day after treatment; (b) there is a good correlation between tumour growth delay and the time for recovery of TFI to the pretreatment level; (c) there is a relationship within a tumour line between the depression of TFI 4 days after treatment and growth delay induced by the same treatment. This relationship appears to be independent of the mechanism by which the agent exerts its cytotoxic effect.**

THE accurate measurement of tumour response to a cytotoxic agent is necessary before we can evaluate the efficacy of individual agents, or combinations of agents. In the laboratory under defined conditions, measurements such as tumour growth delay, *in vitro* clonogenic cell survival (Steel and Adams, 1975), or the proportion of animals cured when treatment is initiated soon after tumour-cell inoculation, have been used to assess quantitatively the value of new and established agents. At present there is no single method of assessment that can be used with absolute confidence with every drug–tumour combination (Connors and Phillips, 1975), but tumour growth inhibition probably serves as the most useful parameter to evaluate drug efficacy in the laboratory.

In the clinic, evaluation of agents becomes far more difficult, and partial or complete tumour regression has been taken to indicate the usefulness of an agent. However, the methods used to measure regression are subject to considerable error (Moertel, 1976) and comparison of results from various clinical trials can to lead to confusion over the clinical value of an agent (Carter and Friedman, 1974). Tumour volume regression *per se* must be regarded with caution, as Steel and Adams (1975) have shown a very small volume regression in a rodent carcinoma after treatment with cyclophosphamide, when the surviving fraction of clonogenic cells was about $10^{-6}$. Clinically, the measurement of tumour growth inhibition may be more sensitive as an indicator of drug effect, but at present is practical for only a few tumour sites.

Many attempts have been made to relate changes in the *in vitro* incorporation of radiolabelled precursors into tumour DNA, to the subsequent patient response. In order to predict the tumour sensitivity in an individual patient, tumour biopsy material has been incubated with various cytotoxic agents, and the drug producing the greatest inhibition of DNA precursor utilization has generally been regarded as potentially the most active against that tumour (Wheeler, Dendy and Dawson, 1974; Wolberg, 1971). Other workers have compared the differential response between the neoplastic biopsy and normal tissue, drug selection being determined by this differential (Tisman, Herbert and Edlis, 1973; Izsak *et al.*, 1971). Alternatively, serial tumour biopsies have been taken after treatment and the radiolabelled precursor utilization *in vitro* compared with that in pretreatment material (Murphy *et al.*, 1975). Several of these studies have suggested that there is a correlation between the depression of DNA synthesis by agents after treatment *in vitro* and a positive clinical response (Livingston *et al.*, 1974; Wheeler *et al.*, 1974; Sky-Peck, 1971).

In previous studies with two transplantable murine tumours (Houghton and Taylor, 1977*a*) we have shown that the time taken *in vivo* for the fractional incorporation of $^3$H-thymidine (TFI) into tumour DNA to recover to its pretreatment level, and the growth delay induced by the same treatment were similar. In this paper, we describe further studies of the relationship between changes in TFI and growth delay in 4 human colonic tumour xenografts, maintained in immune deprived mice, after treatment with various cytotoxic agents. In particular we have considered the following questions: (a) Are tumour growth delay and TFI recovery time (*i.e.* the time required for TFI to return to the pretreatment level) always similar? (b) In different tumour lines, does the same depression of TFI correspond to a similar growth delay following a particular treatment? (c) Is the initial (24-h or 48-h) depression of TFI related to the degree of growth delay produced by different but equally toxic treatments? (d) With a particular tumour line, is there a relationship between the depression of TFI at a given time after treatment and growth delay, which is independent of the mechanism by which the agent kills cells?

### MATERIALS AND METHODS

*Immune deprivation.* — Four-week-old male and female CBA/LAC mice (bred from stock originally supplied by the Laboratory Animal Centre, Carshalton, Surrey) were thymectomized, and 3 weeks later subjected to lethal whole-body $^{60}$Co $\gamma$ radiation (900 rad at 60 rad/min). Within 4 h of irradiation the mice were given $5\times10^6$ syngeneic bone marrow cells suspended in Medium 199, by i.v. injection. These cells were obtained from the femurs and tibias of mice of the same age, thymectomized 3 weeks previously. The animals were used for tumour implantation 2 weeks after the bone marrow transplants.

*Tumour lines.*—The 4 human colonic tumour lines used in this study will be described in greater detail in a subsequent publication (Houghton and Taylor, in preparation). Briefly they constitute the following, listed in decreasing order of differentiation:

HxHC$_1$—a moderately well differentiated adenocarcinoma of the ascending colon, maintained only in female mice.

HxGC$_3$—a poorly differentiated adenocarcinoma of the transverse colon, maintained in male mice.

HxVRC$_5$—a poorly differentiated adenocarcinoma of the caecum, maintained in male mice.

HxELC$_2$—a poorly differentiated carcinoma of the caecum, maintained in male mice.

*Tumour transplantations.*—Tumours were serially transplanted on reaching a diameter of 2 cm, tumours from male and female mice being re-transplanted into the same sex. Pieces approximately 8 mm$^3$ were cut and placed in Medium 199 containing penicillin (200 u/ml) and streptomycin (100 $\mu$g/ml). Four pieces were implanted into the dorsal flanks and in this study over 90% of tumour pieces gave rise to tumours.

*Tumour measurement.*—Caliper measurements of the tumours were initiated about 3 weeks after implantation. Two perpendicular diameters were measured every 3 days and the tumour volume was calculated using the formula Vol$=\pi/6\ d^3$, where $d$ is the mean diameter. Tumours were treated at 8 mm diameter, and growth delay was assessed as the increase in the time required for the treatment tumours to grow to 4× their treatment volume, over the time taken for untreated tumours to show the same growth. The assessment of the induced growth inhibition at 4× the treatment volume allows a sufficient period for the elimination of drug-produced debris. For calculation of growth delay induced by an agent, the change in "relative tumour volume" has been plotted against time after treatment.

The relative tumour volume is the mean cumulative percentage increment (I) in tumour volume, measured every 3 days, and is calculated from the formula:

$$I = \frac{\text{Vol } y - \text{Vol } x}{\text{Vol } x} \times 100.$$

where Vol $y$ is tumour volume 3 days after Vol $x$. The changes in relative volume of individual tumours have been pooled, in some experiments including more than 30 tumours.

*Labelled thymidine fractional incorporation (TFI) assay.*—The measurement of TFI has been described previously (Houghton and Taylor, 1977*a*). Animals were given 25 $\mu$Ci $^3$H-6-TdR (Radiochemical Centre, Amersham, sp. act. 27 Ci/mmol) at various times after administration of the cytotoxic agent, and were killed 1 h later. Tumours were rapidly excised and frozen (−26 °C) until being submitted to a modified Schmidt–Thannhauser extraction (Munro and Fleck, 1968). In these experiments, tumours were weighed and homogenized in KOH (0·3M) and incubated in sealed tubes for 12 h at 37 °C. Samples were cooled (2 °C), neutralized with cold HCl (1M) and acidified to 0·2M with perchloric acid (PCA). After centrifugation, the supernatant was decanted, and the pellet was resuspended in cold PCA (0·2M). After further centrifugation, the supernatants of individual tumours were combined to give the non-incorporated fraction (*i.e.* acid-soluble and RNA) $F_1$, for each individual tumour. DNA was extracted twice by incubating each resuspended pellet in PCA (1M) at 67 °C. The combined DNA extracts constitute the incorporated radioactivity fraction ($F_2$) for each individual tumour. Fractional incorporation is calculated as:

$$\text{TFI} = \frac{F_2 \text{ (ct/min)}}{F_1 + F_2 \text{ (ct/min)}} \times 100$$

Radioactivity was measured by liquid scintillation spectrometry (Intertechnique Ltd, Model SL40) as previously described (Taylor, Tew and Jones, 1976).

In this study, the animals received a single i.p. injection of the cytotoxic agent. The highest dose of each agent used was lethal in 5% of animals ($LD_5$) except in the case of tumour HxGC3 where the highest dose of cyclophosphamide studied was the $LD_{10}$.

### RESULTS

### *TFI in untreated tumours*

Initial studies showed that TFI decreased with increasing tumour mass in each of the 4 xenograft lines studied (Fig. 1). In tumour lines HxELC2 and HxHC1 a plateau was reached at about

FIG. 1.—The TFI in 4 tumour lines plotted against tumour weight. In each case TFI initially decreased during tumour growth.

0·8 g, whereas in tumour lines $HxVRC_5$ and $HxGC_3$ TFI stabilized at about 0·4 g. Consequently, where TFI has been measured after chemotherapy, the result has been expressed as a percentage of that in untreated tumours of equal weight. The reason for this decrease in TFI during tumour growth is not clear, although the results suggest a decrease in growth fraction during the initial "macroscopic" growth period. A decrease in $[^3H]$TdR labelling index has been reported during growth of the Lewis lung rodent carcinoma (Simpson-Herren, Sandford and Holmquist, 1974). However, the most likely explanation for the decreased $[^3H]$TdR utilization during tumour growth is that the precursor penetrates into necrotic zones within the tumour, where it is not incorporated into DNA. The peripheral band of viable tissue maintains a constant thickness during tumour growth (although the actual thickness differs between tumour lines), hence with increasing tumour diameter the proportion of viable to necrotic tissue decreases exponentially. The rate of decrease depends upon the thickness of the viable tissue band. The plateau in TFI may be explained by the radiolabelled precursor being able to penetrate a limited distance into the central necrosis within 1 h of administration. Hence with further tumour growth the ratio of viable to necrotic tissue which the $[^3H]$TdR may penetrate in a given time will remain fairly constant.

*The relationship between early TFI changes and growth delay*

In each of the 4 tumour lines, the depression of TFI at 24 h after drug treatment has been compared with the subsequent growth delay with one of the following agents: cyclophosphamide (CY), 5-fluorouracil (FU), 1–(2–chloroethyl)–3–trans–4–methyl cyclohexyl)–1–nitrosourea (Me CCNU) or actinomycin D (Act D), at equi-toxic levels ($LD_5$). The results are shown in Table I. Although a long growth delay tended to be observed when depression of TFI was greatest, the correlation is not statistically significant ($r = 0·42$ at 13 degrees of freedom). It must be concluded therefore that, neither within nor between series of tumours, is it possible to relate the degree of depression of $[^3H]$-TdR utilization 24 h after treatment with growth delay.

TABLE I.—*The Change in TFI 24 h after Treatment Related to the Mean Growth Delay for the Corresponding Treatment. All Agents were Given at Equitoxic Dose ($LD_5$). — Indicates a Depression in TFI; + Indicates an Increase.*

| Tumour line | Drug | Dose (mg/kg) | Change in TFI (% control) at 24 h | Growth delay (h) |
|---|---|---|---|---|
| $HxHC_1$ | CY | 200 | −21 | none |
| | FU | 200 | +42 | none |
| | Me CCNU | 35 | −40 | 100 |
| | Act D | 0·3 | 0 | none |
| $HxGC_3$ | CY | 200 | −29 | 170 |
| | Fu | 200 | −24 | 30 |
| | Me CCNU | 35 | −47 | 165 |
| | Act D | 0·3 | −65 | 70 |
| $HxVRC_5$ | CY | 200 | −56 | 580 |
| | Fu | 200 | − 3 | 0 |
| | Me CCNU | 35 | −39 | 560 |
| $HxELC_2$ | CY | 200 | −66 | 670 |
| | Fu | 200 | −94 | 1140 |
| | Me CCNU | 35 | −54 | 170 |
| | Act D | 0·3 | −90 | 140 |

*The relationship between TFI recovery time and growth delay*

Our previous study, using two transplantable rodent tumours, showed that the time taken for TFI to recover to the pretreatment level was similar to the duration of tumour growth inhibition for treatment with either CY or $^{60}Co$ radiation (Houghton and Taylor, 1977*a*). The relationship between these two parameters has now been investigated in the 4 human tumour xenograft lines after treatment with various cytotoxic agents.

FIG. 2.—(a) The pattern of TFI in tumour line $HxGC_3$ after various dose levels of CY: △—100; ○—150; ■—200; □—300 mg/kg. Depression is rapid. (b) The TFI recovery time is plotted against dose of CY (mean ± s.e.).

Fig. 2a shows the changes in TFI in tumour line $HxGC_3$ after various doses of CY. It is clear that, 24–48 h after treatment, the depression in TFI is not simply related to the dose of CY. However, when the recovery time is plotted against the corresponding dose of CY (Fig. 2b) there is a clear correlation between them.

Fig. 3 shows the changes in TFI in tumour line $HxVRC_5$ after various doses of CY. At low doses (50 or 100 mg/kg) the TFI recovers to the control level very quickly, whereas at the higher doses (150 and 200 mg/kg) the TFI recovery times are estimated to be 340 h and 600 h respectively. If the hypothesis that the TFI recovery time correlates with growth delay (rather than the level of the initial depression) is correct, little or no growth delay would be expected at low doses in this tumour, whereas at higher doses (150 and 200 mg/kg) considerable growth delay ought to be observed. Growth curves for tumour $HxVRC_5$ after treatment with various doses of CY are presented in Fig. 4a. The growth curves for the other tumour lines in Fig. 4 show that after treatment, even at doses inducing considerable growth inhibition, no volume regressions were found. In most cases, tumour growth continued at a reduced rate, but appeared to recover to that of untreated tumours (of the same line and weight) by the time they had grown to 4 times the treatment volume (marked by a horizontal arrow in Fig. 4).

Fig. 5 shows the relationship between TFI recovery time and growth delay for the corresponding dose of CY in tumour

FIG. 3.—The pattern of TFI in tumour line $HxVRC_5$ after various dose levels of CY: △—50; ○—100; ■—150; □—200 mg/kg. TFI is expressed as a percentage of the level measured in untreated tumours of the same weight (mean ± s.e.).

FIG. 4.—Tumour growth curves following various treatment: (a) $HxVRC_5$ after CY; (b) $HxGC_3$ after CY; (c) $HxELC_2$ after CY and (d) $HxELC_2$ after FU (note change of scale). Figures above each curve refer to the dose (mg/kg) of agent administered (Co = Control). Treatment at the vertical arrow, and growth delay assessed at the horizontal arrow.

FIG. 5.—In tumour line HxELC$_2$ the response to CY as measured by growth delay (□) or TFI recovery time (■) was dose-dependent. There appears to be a threshold dose (16 mg/kg) below which there is no response to CY.

line HxELC$_2$. In general, the TFI recovery time and growth delay for the same treatment are similar (Table II). The data in Table II show the TFI recovery time and the mean growth delay for the 36 tumour : drug–dose combinations examined to date, and indicate a marked correlation between the two measurements ($r = 0{\cdot}96$). It is of interest that only 1 of the 4 human colonic xenografts (HxELC$_2$) showed a prolonged depression of TFI after FU treatment. The other tumour lines each showed an increased uptake and utilization of this "salvage" DNA precursor, and this ability to utilize pre-formed TdR could account for their relative insensitivity to FU (Houghton, Houghton and Taylor, 1977*b*, 1977*c*).

*The relationship between depression of TFI and growth delay at one time after treatment within a tumour line*

It was shown above that it is not possible to relate a given depression in TFI 24 h after treatment to growth delay. Similarly, with the depression in TFI 50 h after various doses of CY in 3 xenograft lines, it is clear that, although there may be a relationship between the degree of depression of TFI and its recovery time, and growth delay within a tumour line, this relationship is unique to each line (Table III). Consequently, an agent inducing a similar TFI depression in two individual tumour lines at a particular

TABLE II.—*The TFI Recovery Time for 4 Tumour Lines after Treatment with Various Agents, Compared to the Growth Delay. There appears to be Considerable Agreement between the Two Measurements. $r = 0{\cdot}95$ for the 36 Drug/Tumour Combinations)*

| Tumour line | Passage | Agent | Dose (mg/kg) | TFI recovery time (h) | Growth delay (h) |
|---|---|---|---|---|---|
| HxHC$_1$ | 11 | Act D | 0·075 | 0 | 0 |
| | | | 0·15 | 0 | 0 |
| | | | 0·3 | 0 | 0 |
| | 12 | MeCCNU | 17·5 | 90 | ND* |
| | | | 35 | 120 | 100 |
| | 12 | Cis-DDP† | 3 | 0 | 0 |
| | | | 6 | 45 | ND |
| | 7 | CY | 50 | 0 | 0 |
| | | | 100 | 50 | 0 |
| | | | 200 | 80 | 0 |
| | 7 | FU | 100 | 0 | 0 |
| | | | 200 | 0 | 0 |
| HxGC$_3$ | 9 | Act D | 0·3 | 70 | 70 |
| | | | 0·5 | 150 | 170 |
| | 9 | MeCCNU | 35 | 150 | 165 |
| | 5 | Cis-DDP | 3 | 0 | ND |
| | 8 | | 6 | 30 | ND |
| | 5 | CY | 100 | 50 | 70 |
| | | | 150 | 80 | ND |
| | | | 200 | 110 | 170 |
| | | | 300 | 190 | 250 |
| | 5 | FU | 50 | 0 | 0 |
| | | | 100 | 0 | 0 |
| | | | 150 | 35 | 0 |
| | | | 200 | 60 | 30 |
| HxVRC$_5$ | 12 | MeCCNU | 35 | 550 | 560 |
| | 6 | CY | 50 | 20 | 0 |
| | | | 100 | 60 | 70 |
| | | | 150 | 340 | 290 |
| | | | 200 | 600 | 580 |
| | 6 | FU | 50 | 0 | 0 |
| | | | 100 | 0 | 0 |
| | | | 150 | 0 | ND |
| | | | 200 | 0 | 0 |
| HxELC$_2$ | 5 | MeCCNU | 17·5 | 70 | ND |
| | | | 35 | 150 | ND |
| | 5 | CY | 50 | 100 | 130 |
| | | | 100 | 340 | 320 |
| | | | 150 | 480 | ND |
| | | | 200 | 700 | 670 |
| | 5 | FU | 50 | 80 | ND |
| | | | 100 | 300 | 450 |
| | | | 150 | 500 | ND |
| | | | 200 | 900 | 1140 |
| | 5 | Act D | 0·075 | 30 | 0 |
| | | | 0·15 | 40 | 70 |
| | | | 0·3 | 80 | 140 |

* ND = Not determined.
† Cis-DDP = cis-platinum (II) diamine dichloride.

TABLE III.—*Per Cent Reduction in TFI in 3 Xenograft Tumour Lines 50 h after CY Treatment Compared to the Corresponding TFI Recovery Time and Mean Growth Delay Produced. It is Shown that for a Given Level of TFI Depression, the Recovery Time and Growth Delay may Differ Considerably between Tumour Lines*

| Tumour line | CY dose (mg/kg) | % Reduction in TFI 50 h after treatment | Mean TFI recovery time (h) | Mean growth delay (h) |
|---|---|---|---|---|
| $HxGC_3$ | 300 | $82\pm1$ | 190 | 250 |
| | 200 | $78\pm2$ | 110 | — |
| | 100 | $4\pm10$ | 50 | 70 |
| $HxVRC_5$ | 200 | $52\pm4$ | 600 | 580 |
| | 150 | $42\pm1$ | 340 | 290 |
| | 100 | $15\pm3$ | 60 | 70 |
| $HxELC_2$ | 200 | $97\pm1$ | 700 | 670 |
| | 100 | $52\pm5$ | 340 | 320 |
| | 50 | $30\pm4$ | 100 | 130 |

time after treatment may delay the growth of each tumour line quite differently. However, within a tumour line there may be a relationship between depression of TFI and growth delay that is independent of the mechanism by which the agent kills tumour cells (Houghton and Taylor, 1977*a*). Our previous experiments using rodent tumours suggested that such a relationship may be established at some time after the nadir of TFI

FIG. 6.—In tumour line $HxELC_2$ results from FU- and CY-treated animals have been combined to establish a relationship between TFI depression 100 h after treatment, and other tumour responses. (a) TFI depression plotted against TFI recovery time for tumours receiving the same treatment. (b) TFI depression plotted against mean growth delay for tumours receiving the same treatment. (Results are mean $\pm$s.e.).

depression. The relationship between depression in TFI 4 days after treatment and TFI recovery time or growth delay in tumour line $HxELC_2$ following FU and CY treatment is shown in Fig. 6 (a) and (b). The data show that there is a significant correlation between the depression of TFI at 4 days after treatment, the TFI recovery time and the growth delay in this tumour line. It has not been possible in this study to determine whether similar relationships exist for the other 3 tumour lines studied, since these lines were not sufficiently sensitive to the drugs listed to enable meaningful relationships to be established.

### DISCUSSION

There appears to be general agreement between mean growth delay and mean TFI recovery time for the corresponding treatment in this series of human colonic xenografts. In each of the tumour–drug combinations we have studied the post-treatment growth rate has eventually returned to that of the untreated control. From this point onward, whether drug-induced growth delay was assessed at 4, 6 or 8 times the treatment volume, the result was the same (*i.e.* control and treated tumour growth curves became parallel). It would appear, therefore, that the TFI recovery time corresponds closely to the time at which the treated tumour regains the same growth rate as that measured in untreated tumours of the same weight. If changes in TFI were to parallel those in "clonogenic cells" following treatment, it would be expected that TFI would be depressed below detectable levels for some time, followed by a rapid recovery to the pretreatment level, with a doubling time similar to that of the repopulating cells. At times soon after treatment, TFI is almost certainly influenced by [$^3$H]-TdR incorporation into doomed cells, and the shape of the TFI recovery curve is probably determined by the influx of cells into the proliferative cycle and the efflux of drugkilled cells which cease to proliferate after a few days.

Several studies have attempted to predict individual patient response by examining the depression of radiolabelled DNA precursor incorporation in biopsy samples incubated with a selection of cytotoxic agents *in vitro*. The data presented here show clearly that, 24 h after treatment, it is not possible to predict tumour growth delay from changes in TFI either within a group of human colonic xenografts, or even within one particular tumour line. Further, the agent producing the greatest TFI 24-h depression within a tumour line may not induce the greatest growth inhibition. Consequently, it is not possible to select the most effective agent by measurement of [$^3$H]TdR incorporation 24 h after treatment. However, in our study, if there was no depression in TFI at 24 h, no growth delay was observed.

In the 4 tumour lines studied in this work, there is a clear relationship between growth delay and TFI recovery time ($n=36$, $r=0{\cdot}96$). In one tumour line (HxELC$_2$) we have been able to establish a relationship between TFI depression 4 days after treatment and growth delay ($r=0{\cdot}96$) or TFI recovery time ($r=0{\cdot}86$). The time at which such a relationship is established will probably vary with each tumour line, but "calibration" of a tumour line in this manner may allow rapid evaluation of single agents, or combinations of agents.

It is not certain whether any of the tumour-inhibitory effects presented in this study would be regarded as positive tumour responses in the clinic, for in each case the tumours have continued to grow after treatment, and the criterion of a 50% reduction in the product of the tumour diameters has not been satisfied. However, that tumour growth inhibition has been induced (Fig. 4a–d) is apparent when the growth curves for treated tumours are compared with those for untreated tumours.

Our data show that the TFI recovery time correlates with growth delay produced by the same treatment, and in the laboratory, where tumour lines may be "calibrated", the assay may allow rapid assessment of the value of drug combinations and schedules. However, the data show that changes in utilization of [$^3$H]TdR by tumours soon after the administration of cytotoxic agents are a poor indicator of tumour response *in situ*, and such results obtained 1 day after treatment must be regarded with caution.

### REFERENCES

Carter, S. K. & Friedman, M. (1974) Integration of Chemotherapy into Combined Modality Treatment of Solid Tumours. II. Large Bowel Carcinoma. *Cancer Treatment Rev.*, **1**, 111.

Connors, T. A. & Phillips, B. J. (1975) Screening for Anticancer Agents; the Relative Merits of *In vitro* and *In vivo* Techniques. *Biochem Pharmac.*, **24**, 2217.

Houghton, P. J. & Taylor, D. M. (1977*a*) Fractional Incorporation of 3H-thymidine and DNA Specific Activity as Assays of Inhibition of Tumour Growth. *Br. J. Cancer*, **35**, 68.

Houghton, P. J., Houghton, J. A. & Taylor, D. M. (1977*b*) Factors Determining the Response to 5-fluorouracil in Human Colonic Tumour Xenografts (Abstract). *Br. J. Cancer* **36** (in press).

Houghton, J. A., Houghton, P. J. & Taylor, D. M. (1977*c*) The Physical and Biochemical Responses of Four Human Colon Xenografts Following 5-fluorouracil Treatment. *Eur. J. Cancer* (Submitted).

Izsak, F. C., Eylan, E., Gazith, A., Shapiro, J., Naharin, S. & Raanani, C. (1971) Growth Inhibiting Effects of Cytotoxic Agents on Human Tumour and Tumour-bearing Normal Tissue *In vitro. Eur. J. Cancer*, **7**, 33.

Livingston, R. B., Ambus, U., George, S. L., Freireich, E. J. & Hart, J. S. (1974) *In vitro* Determination of Thymidine-3H Labelling Index in Human Solid Tumours. *Cancer Res.*, **34**, 1376.

Moertel, C. G. (1976) The Role of Chemotherapy and Immunology in the Management of upper gastrointestinal tract Cancer. *Br. J. Cancer*, **34**, 325.

Munro, H. N. & Fleck, A. (1968) The Determination of Nucleic Acids. *Meth. biochem. Analysis*, **14**, 113.

Murphy, W. K., Livingston, R. B., Ruiz, V. G., Gercovich, F. G., George, S. L., Hart, J. S. & Freireich, E. J. (1975) Serial Labelling Index Determination as a Predictor and Response in Human Solid Tumours. *Cancer Res.*, **35**, 1438.

Simpson-Herren, L., Sandford, A. H. & Holmquist, J. P. (1974) Cell Population Kinetics of Transplanted and Metastatic Lewis Lung Carcinoma. *Cell Tissue Kinet.*, **7**, 349.

Sky-Peck, H. H. (1971) Effects of Chemotherapy on the Incorporation of 3H-Thymidine into DNA of Human Neoplastic Tissue. *Natl Cancer Inst. Monograph*, **34**, 197.

Steel, G. G. & Adams, K. (1975) Stem Cell Survival and Tumour Control in the Lewis Lung Carcinoma *Cancer Res.*, **35**, 1530.

Taylor, D. M., Tew, K. D. & Jones, J. D. (1976) Effects of cis-Dichlorodiammine Platinum (II) on DNA Synthesis of Normal and Tumour-bearing Rats. *Eur. J. Cancer*, **12**, 249.

Tisman, G., Herbert, V. & Edlis, H. (1973) Determination and Therapeutic Index of Drugs by *In vitro* Sensitivity Tests using Human Host and Tumour Cell Suspensions. *Cancer Chemotherapy Rep.*, **57**, 11.

Wheeler, T. K., Dendy, P. P. & Dawson, A. (1974) Assessment of an *In vitro* Screening Test of Cytotoxic Agents in the Treatment of Advanced Malignant Disease. *Oncology*, **30**, 362.

Wolberg, W. H. (1971) Biochemical Approaches to Prediction of Response in Solid Tumours. *Natl Cancer. Inst. Monograph*, **34**, 189.